# Histone H1-mediated epigenetic regulation controls germline stem cell self-renewal by modulating H4K16 acetylation

**DOI:** 10.1038/ncomms9856

**Published:** 2015-11-19

**Authors:** Jin Sun, Hui-Min Wei, Jiang Xu, Jian-Feng Chang, Zhihao Yang, Xingjie Ren, Wen-Wen Lv, Lu-Ping Liu, Li-Xia Pan, Xia Wang, Huan-Huan Qiao, Bing Zhu, Jun-Yuan Ji, Dong Yan, Ting Xie, Fang-Lin Sun, Jian-Quan Ni

**Affiliations:** 1 School of Medicine, Tsinghua University, Beijing 100084, China; 2 Research Center for Translational Medicine at East Hospital, School of Life Sciences and Technology, Tongji University, Shanghai 200092/200120, China; 3 College of Bioengineering, Hubei University of Technology, Wuhan 430068, China; 4 Tsinghua Fly Center, Tsinghua University, Beijing 100084, China; 5 Institute of Biophysics, Chinese Academy of Sciences, Beijing 102206, China; 6 Department of Molecular and Cellular Medicine, College of Medicine, Texas A&M Health Science Center, College Station, Texas 77843, USA; 7 Department of Genetics, Harvard Medical School, Boston, Massachusetts 02115, USA; 8 Stowers Institute for Medical Research, Kansas City, Missouri 64110, USA; 9 Department of Anatomy and Cell Biology, University of Kansas School of Medicine, Kansas City, Kansas 66160, USA

## Abstract

Epigenetics plays critical roles in controlling stem cell self-renewal and differentiation. Histone H1 is one of the most critical chromatin regulators, but its role in adult stem cell regulation remains unclear. Here we report that H1 is intrinsically required in the regulation of germline stem cells (GSCs) in the *Drosophila* ovary. The loss of H1 from GSCs causes their premature differentiation through activation of the key GSC differentiation factor *bam*. Interestingly, the acetylated H4 lysine 16 (H4K16ac) is selectively augmented in the H1-depleted GSCs. Furthermore, overexpression of *mof* reduces H1 association on chromatin. In contrast, the knocking down of *mof* significantly rescues the GSC loss phenotype. Taken together, these results suggest that H1 functions intrinsically to promote GSC self-renewal by antagonizing MOF function. Since H1 and H4K16 acetylation are highly conserved from fly to human, the findings from this study might be applicable to stem cells in other systems.

## Abstract (web summary)

Epigenetics plays critical roles in controlling stem cell self-renewal and differentiation. Here, Sun *et al.* show that H1 is intrinsically required in the regulation of germline stem cells in the *Drosophila* ovary by antagonizing MOF, a histone acetyltransferase specific for H4K16.

## Graphical abstract

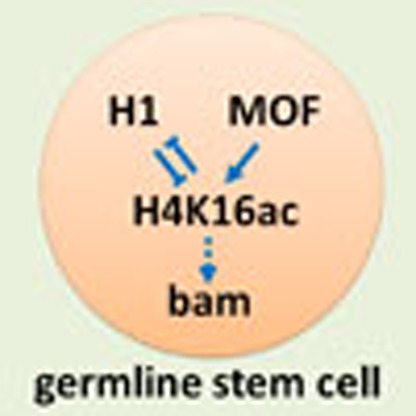

Chromatin is a complex of DNA and proteins within the eukaryotic cell nucleus. The nucleosome is the basic unit of chromatin and consists of five highly positively charged histones called linker histone H1 (H1), H2A, H2B, H3 and H4. The core of the nucleosome octamer contains one tetramer of H3 and H4 and two dimers of H2A and H2B^1^, which undergo a variety of post-translational modifications. These modifications collectively influence the local chromatin structure and correlate with gene transcription^2^,^3^, and are tightly associated with stem cell self-renewal, differentiation and proliferation^4^,^5^. H1 is also believed to be critical for chromatin remodelling through the condensation of nucleosomes^6^,^7^,^8^,^9^. In mammals, H1 controls chromatin dynamics during early embryogenesis^10^, with just a 50% reduction in H1 variants causing embryonic lethality with a broad array of phenotypes^11^. In addition, the depletion of H1 variants could directly block the differentiation of mouse embryonic stem cells^12^. However, the role of H1 in the regulation of adult stem cells remains to be determined.

One of the major challenges in elucidating the developmental roles of H1 *in vivo* is the high degree of heterogeneity, as multiple H1 variants with redundant functions exist in most species^6^,^13^,^14^. With only one version of H1 expressed post-embryonically^15^ and well-defined stem cells^16^, the *Drosophila* ovary is a particularly attractive system to study the functions of H1 in adult stem cell regulation (Supplementary Fig. 1a). In the *Drosophila* ovary, two or three germline stem cells (GSCs) are located at the anterior end of the germarium, which is situated at the tip of each ovariole. They physically interact with cap cells anteriorly and escort cells (ECs) laterally. GSCs can be recognized by their proximity to the cap cells and the presence of a spherical organelle known as the spectrosome^17^. The immediate differentiating GSC daughters, cystoblasts, also carry a spectrosome, but are distant from cap cells. Further differentiated germ cell cysts contain a branched fusome, which is an identical organelle to the spectrosome with a different morphology^17^. Therefore, GSCs and their differentiated daughters can be followed and studied.

GSC self-renewal is known to be co-ordinately regulated by both extrinsic signals from niche cells and intrinsic factors 16. In the adult *Drosophila* ovary, cap cells and anterior ECs form a self-renewal niche^18^,^19^. GSCs receive extrinsic signals such as Decapentaplegic (Dpp), the *Drosophila* homologue of the vertebrate bone morphogenetic proteins for their maintenance^20^. Dpp signalling preserves GSC characteristics and suppresses the transcription of *bag-of-marbles* (*bam*), which is both necessary and sufficient for the differentiation of the early GSC lineage^21^,^22^. Chromatic remodelling factors, ISWI and Domino, have also shown to act intrinsically to promote GSC self-renewal by preventing differentiation, indicating that epigenetic regulation is important intrinsically for controlling GSC self-renewal^23^,^24^. In addition, Bre-containing protein complex and Enok are also required intrinsically to maintain GSC self-renewal^25^,^26^. Another potential chromatin regulator Stonewall is also involved in the regulation of GSC fate^4^. Various other intrinsic factors, including Mad, Piwi, Scrawny and Eggless, are also important to maintain GSC self-renewal^16^.

In this study, we have first revealed a critical role of H1 in maintaining GSCs by preventing *bam* activation. Interestingly, we find that the H4K16ac level is selectively upregulated in the H1 knockdown GSCs, and that the association of H1 on chromatin is antagonized by males absent on the first (MOF), a histone acetyltransferase specific for H4K16. Most interestingly, the knocking down of *mof* significantly suppresses the GSC loss phenotype induced by the depletion of H1. Taken together, we find that a balance between H1 and H4K16ac in the chromatin is required for the maintenance of GSCs.

## Results

### H1 is intrinsically required for the maintenance of GSCs

**Figure 1:**
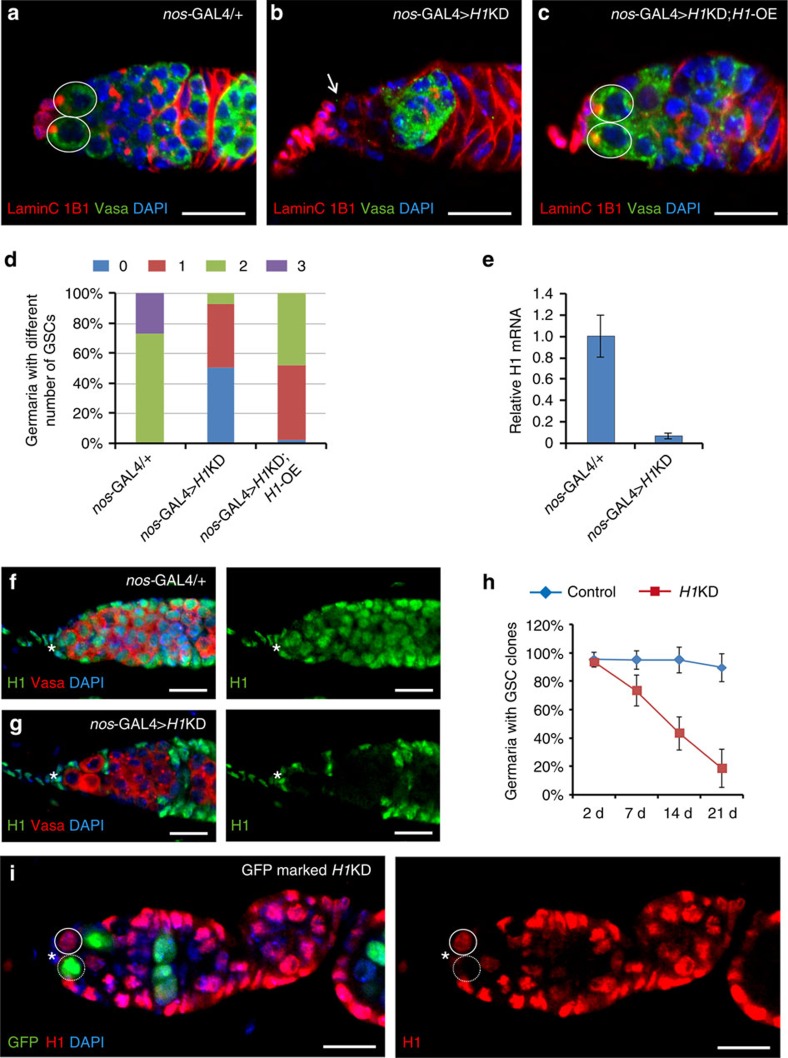
H1 is required for GSC maintenance intrinsically. (**a**–**c**) Germaria are from 3-day-old adult flies, and are stained with 1B1 (red) to reveal spectrosomes/fusomes, anti-Vasa (green) to show the germ cells, 4,6-diamidino-2-phenylindole (DAPI, blue) to show the nuclei and anti-LaminC (also red) to show the nuclear lamina of cap cells and terminal filament cells. Germaria from controls (**a**) have two GSCs (circles), whereas those from *nos*-GAL4>*H1*KD (**b**) have no GSC (arrow). (**c**) *H1*-OE rescues the loss of GSCs. (**d**) Column chart showing that *nos*-GAL4>*H1*KD germaria contain fewer GSCs than controls, and that *H1*-OE can rescue the *H1*KD GSC loss phenotype. *n*=99, 97 and 90 for the three groups, respectively. (**e**) qRT–PCR results show that the transcription of H1 in *nos*-GAL4>*H1*KD ovaries is reduced to 7.4% compared with that in the control. Data are shown as mean±s.d. of three independent experiments. (**f**,**g**) H1 (green) is reduced to undetectable level in *nos*-GAL4>*H1*KD germline cells (**g**) compared with that in the control (**f**), in 3-day-old adults. (**h**) The percentage of germaria carrying *H1*KD GSC clones dramatically decreases with time compared with those carrying wild-type GSC clones. Results are from three experiments and the error bars show the 95% confidential intervals. (**i**) H1 is eliminated in GFP-marked *H1*KD GSCs (broken circles) compared with that in GFP-negative GSCs (circles) 7 days ACI. Scale bars, 10 μm. Anterior is to the left in this and all subsequent images of germaria. Asterisks indicate the niche cap cells in this and all subsequent images. d, day.

To overcome the challenge of genetic analysis of H1 due to its multiple gene copies in the *Drosophila* genome, we took advantage of the transgenic RNA interference (RNAi) method by targeting the shared coding sequence of the H1 genes. We generated a transgenic RNAi line (*H1*KD line) using a recently developed approach that allowed for knocking-down of H1 in both spatial and temporal manners^27^. In combination with a *nanos*-GAL4 (*nos*-GAL4) line that specifically expresses GAL4 in germ cells (Supplementary Fig. 1c,d), all *H1*KD females (*nos*-GAL4>*H1*KD) failed to lay any eggs, indicating that H1 is required for oogenesis. As neither the heterozygous *nos*-GAL4 flies (*nos*-GAL4/+) nor the *nos*-GAL4-driven green fluorescent protein (GFP) RNAi flies (*nos*-GAL4>*GFP*-KD) showed any defects in GSC maintenance (Supplementary Fig. 1e–i), we used these two genotypes interchangeably as controls for subsequent experiments. In each control germarium from 3-day-old adults, there were two to three round spectrosome-containing GSCs, recognized by 1B1 antibody staining (Fig. 1a,d). In contrast, 92% of *nos*-GAL4>*H1*KD germaria retained only one or zero stem cell immediately adjacent to the cap cells (Fig. 1b,d). We validated the efficiency of H1 depletion using quantitative reverse transcription–PCR (qRT–PCR), immunostaining and western blot (Fig. 1e–g, Supplementary Fig. 3a,b). As shown in Fig. 1e, the H1 transcript level in *nos*-GAL4>*H1*KD ovaries had reduced to 7.4% of that in the controls. The qRT–PCR result was further confirmed using another set of primers (Supplementary Fig. 3b), and was consistent with the western blot assay (Supplementary Fig. 3a). By immunostaining with an H1 specific antibody^28^, H1 protein was shown to be efficiently and specifically depleted in the germline of *nos*-GAL4>*H1*KD flies, but not in the somatic cells (Fig. 1f,g). To further validate that the GSC loss phenotype is indeed caused by H1 depletion, we generated two additional transgenic RNAi lines targeting different sections of the coding regions of H1 and observed similar GSC loss phenotypes when they are expressed using *nos*-GAL4 (Supplementary Fig. 2d–h). Furthermore, the expression of H1 cDNA that is not sensitive to RNAi significantly rescued the GSC loss phenotype caused by *H1*KD (Fig. 1c,d and Supplementary Fig. 2a–c). Taken together, these observations demonstrate that H1 is required intrinsically for GSC maintenance.

As dBigH1 is also expressed in the *Drosophila* germline^15^, the question is raised as to whether dBigH1 is involved in the GSC maintenance phenotype we observed with H1 knockdown. We performed qRT–PCR using *nos*-GAL4>*H1*KD fly ovaries, and did not detect a significant decrease in dBigH1 expression (Supplementary Fig. 3a,b and Supplementary Fig. 3h,i). Furthermore, we constructed two independent dBigH1 transgenic RNAi fly lines, which efficiently knocked down dBigH1 expression when driven by *nos*-GAL4 (Supplementary Fig. 3c), but did not observe any GSC maintenance phenotypes (Supplementary Fig. 3d–f). In addition, antibody staining showed that both dBigH1 and H1 were expressed in the germline but in distinct patterns, as dBigH1 was mostly expressed at later stages of oogenesis, and in the oocyte (Supplementary Fig. 3h) and nurse cells proximal to the oocyte^15^, whereas H1 was expressed in early-stage germline cells, but not in the oocyte and at a decreased level in nurse cells neighbouring the oocyte (Supplementary Fig. 3g). These results suggest that H1's roles in GSC maintenance do not involve dBigH1.

To determine further if H1 is required in adult GSCs for their maintenance, we generated *H1*KD clones in GSCs using a conditional germline FLP-out system^29^ (Supplementary Fig. 1b). This approach allowed us to deplete H1 in adult germline cells marked by GFP after clone induction (ACI; Fig. 1i). In the controls, the percentage of germaria with GFP-positive GSCs remained at a similar level 3 weeks ACI compared with that at 2 days ACI (Fig. 1h). However, the percentage of germaria with GFP-marked GSCs remarkably declined to 19% 3 weeks ACI (Fig. 1h). These results support the idea that H1 is intrinsically required for the maintenance of adult GSCs.

### Intrinsic depletion of H1 leads to *bam* activation in GSCs

At the cellular level, the H1 depletion-induced GSC loss phenotype could be caused by apoptosis, premature differentiation, or both. Previous studies showed that H1 depletion can trigger genomic instability and apoptosis in somatic cells *in vivo* and in cultured embryonic cells^30^,^31^,^32^,^33^. However, we did not observe apoptotic GSCs in the *H1*KD germaria by anti-cleaved caspase-3 immunostaining (Supplementary Fig. 4a). Furthermore, H1 knockdown cysts have been detected in egg chambers (Fig. 1i and Supplementary Fig. 3i), which is consistent with the apoptosis assay and suggests that H1 is not required after the germline cells have differentiated. In addition, the GSC loss phenotype was not rescued upon expression of the caspase inhibitor p35 (Supplementary Fig. 4b–d). These results suggest that the GSC loss phenotype caused by H1 depletion is unlikely due to apoptosis.

**Figure 2:**
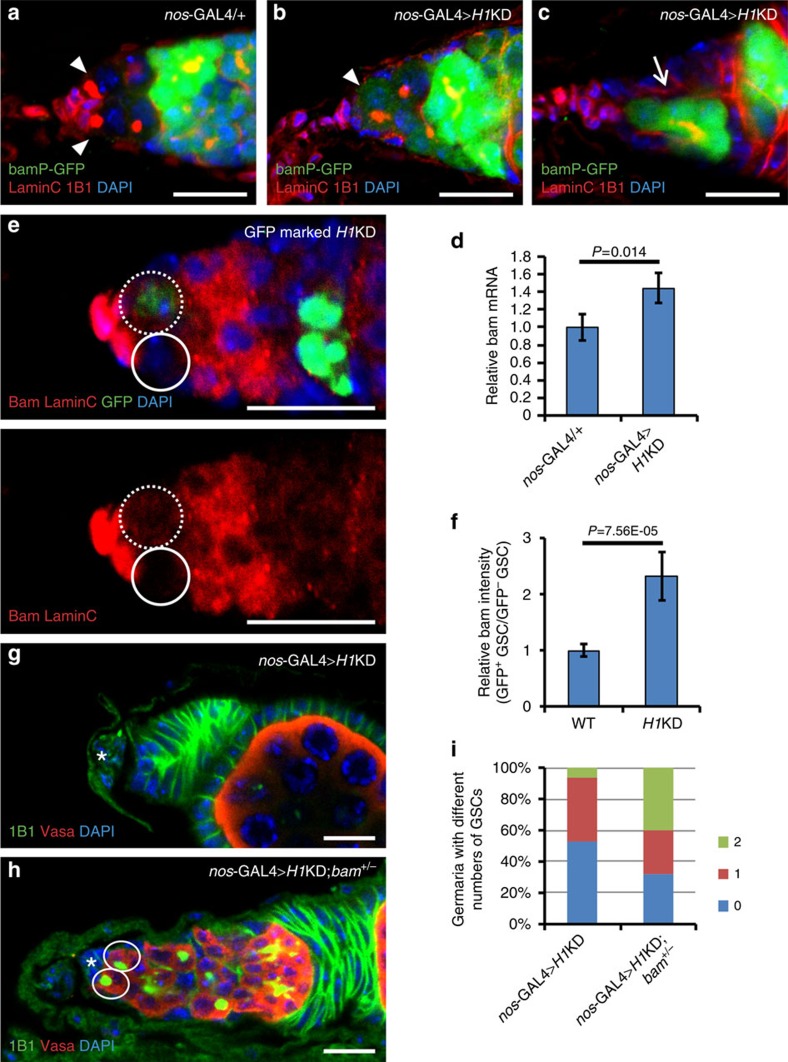
The loss of *H1*KD GSCs is due to premature differentiation triggered by *bam* upregulation. (**a**–**c**) bamP-GFP expression (green) is repressed in GSCs of control flies (**a**), but expands to the GSCs in *nos*-GAL4>*H1*KD germaria (arrowhead in **b**). Note that the bamP-GFP-positive germline cells (arrow) neighbouring the cap cells (red nuclear lamina) in **c** share a branched fusome (red), indicating that premature differentiation of the GSCs occurs in *H1*KD germaria. Arrowheads in **a** point to GSCs. (**d**) qRT–PCR results show that the transcription of *bam* in *nos*-GAL4>*H1*KD ovaries has increased to 1.5-fold compared with that in the control (*n*=3, mean±s.d.). (**e**) Bam (red) is increased in the GFP-marked *H1*KD GSCs (broken circles) compared with that in the GFP-negative control GSCs (circles) 7 days ACI. (**f**) Quantification of results in **e** showing that the relative Bam intensity in GFP-marked *H1*KD GSCs is about twofold higher than that in GFP-negative control GSCs (*n*=5, mean±s.d.). The intensity of Bam in control GSCs is set to 1. Data are evaluated with Student's *t*-test. (**g**,**h**) The *bam*^*Δ86*^ heterozygous mutation (**h**) can suppress GSC loss in *nos*-GAL4>*H1*KD females (**g**). (**i**) Quantification of results in **g**,**h** showing that the *bam*^*Δ86*^ heterozygous mutation can rescue the GSC reduction in *H1*KD germaria. *n*=61 and *n*=25 for *nos*-GAL4>*H1*KD and *nos*-GAL4>*H1*KD; *bam*^*+/−*^ groups, respectively. Scale bars, 10 μm. DAPI, 4,6-diamidino-2-phenylindole.

To test whether H1 depletion causes the premature differentiation of GSCs, we analysed the transcription activation of *bam* in the H1-depleted GSCs, using bamP-GFP as a reporter^22^. As demonstrated earlier, ectopic expression of *bam* can trigger GSC premature differentiation, and therefore result in GSC loss^34^. In control germaria, bamP-GFP accumulated primarily in late cystoblasts and differentiating germline cells and was undetectable in the GSCs (Fig. 2a). However, in *H1*KD germaria, bamP-GFP expression was not only observed in differentiating germline cells but also in GSCs and germline cysts attached to the niche (Fig. 2b,c), suggesting that premature differentiation of GSCs likely contributes to their loss when H1 is depleted. In addition, qRT–PCR experiments showed that the *bam* expression level in *nos*-GAL4>*H1*KD ovaries had increased to 1.5-fold of that in the control ovaries (Fig. 2d). Furthermore, we examined the endogenous protein level of Bam in the *H1*KD mosaic germarium using immunostaining. In contrast to control GSCs, the intensity of the Bam signal was increased in *H1*KD GSCs (Fig. 2e,f), further supporting that the ectopic expression of *bam* after H1 depletion is associated with GSC loss.

Next, we asked whether the upregulation of *bam* in *H1*KD GSCs is caused by compromised Dpp signalling. We examined the expression levels of Dad-lacZ and pMad, two reporters of Dpp signalling activity^20^,^35^, in the *H1*KD mosaic germaria. Both Dad-lacZ and pMad were expressed in GFP-positive GSC clones with indistinguishable intensities and patterns 1 week ACI (Supplementary Fig. 4e,f), compared with those in the GFP-negative control GSCs. Therefore, it is likely that H1 functions downstream of Dpp signalling to repress *bam* in GSCs.

To test further whether Bam upregulation is the major cause of the stem cell phenotypes in *H1*KD germaria, we introduced one copy of *bam*^*Δ86*^, a mutant allele of *bam*, into the *H1*KD GSCs. The *bam*^*Δ86*^ heterozygous ovaries show a moderate accumulation of GSC-like cells^36^. As expected, the presence of one copy of *bam*^*Δ86*^ significantly rescued the loss of GSCs in *nos*-GAL4>*H1*KD ovaries, with 40% of germaria carrying two GSCs compared with 6.5% of those with two wild-type *bam* alleles (Fig. 2g–i). Taken together, these results support the notion that H1-induced activation of *bam* expression is largely responsible for the loss of GSCs in *H1*KD ovaries.

### *H1*KD selectively results in hyperacetylation on H4K16

**Figure 3:**
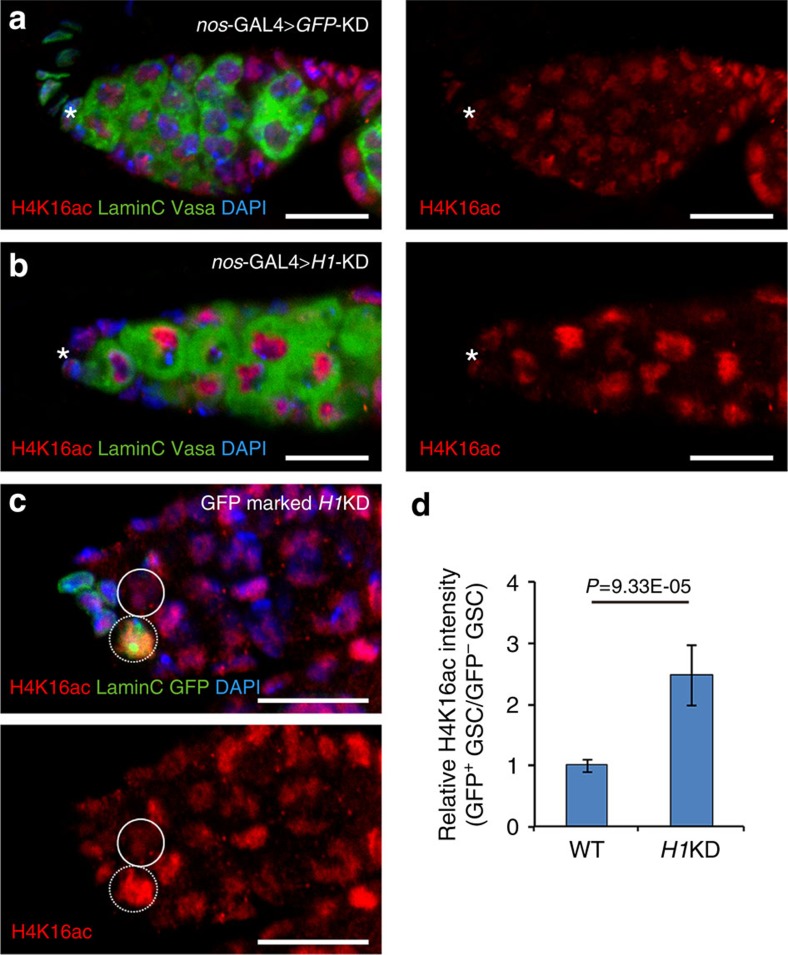
H1 selectively modulates H4K16ac level. (**a**,**b**) H4K16ac levels (red) in the germline are increased in *nos*-GAL4>*H1*KD flies (**b**) compared with those in controls (**a**). Anti-Vasa antibody (green; cytoplasm) marks the germline cells, and anti-LaminC (also green; nuclear lamina) marks the cap cells and terminal filaments. (**c**) H4K16ac level (red) in the GFP-positive (nucleus) *H1*KD GSC (broken circles) is markedly elevated compared with that in the GFP-negative control GSC (circles). Anti-LaminC (also green; nuclear lamina) marks the cap cells and terminal filaments. (**d**) Quantification of results in **c** showing that the relative H4K16ac fluorescent intensity in the GFP-positive *H1*KD clone is more than twofold higher compared with that in the GFP-negative control GSCs (*n*=5, mean±s.d.). The intensity of H4K16ac in control GSCs is set to 1. Data are evaluated with Student's *t*-test. Scale bars, 10 μm. DAPI, 4,6-diamidino-2-phenylindole.

Loss of H1 has been shown to alter several histone modifications, including the methylation of H3K4, H3K9 and H3K27, in specific genes during embryonic stem cell differentiation *in vitro*^12^. We examined various histone modifications in *H1*KD germaria driven by *nos*-GAL4, including the transcriptional repression ones (H3K9me2 and H3K27me3) and the transcriptional activation ones (H3K4me2, H4K5ac, H4K8ac, H4K16ac and H4K12ac). Interestingly, the level of H4K16ac, a hallmark of hyperactive chromatin, was distinctly upregulated in the germline cells upon depletion of H1 (Fig. 3a,b), but the other modifications remained unchanged (Supplementary Fig. 5a–l). Using a mosaic analysis in GSCs, we confirmed the upregulation of H4K16ac signals in *H1*KD GSCs (Fig. 3c,d). The augmented H4K16ac is not due to upregulation of *mof*, a specific H4K16 acetyltransferase^37^,^38^, as the expression level of *mof* was not affected upon H1 depletion (Supplementary Fig. 4g). In addition, the increased H4K16ac was not due to upregulation of H4 level, as the levels of H4K5ac, H4K8ac and H4K12ac were not altered in GSCs compared with those in the control. Therefore, these results suggest that H1 is required to maintain appropriate levels of H4K16ac in GSCs.

### MOF antagonizes the association of H1 on chromatin

**Figure 4:**
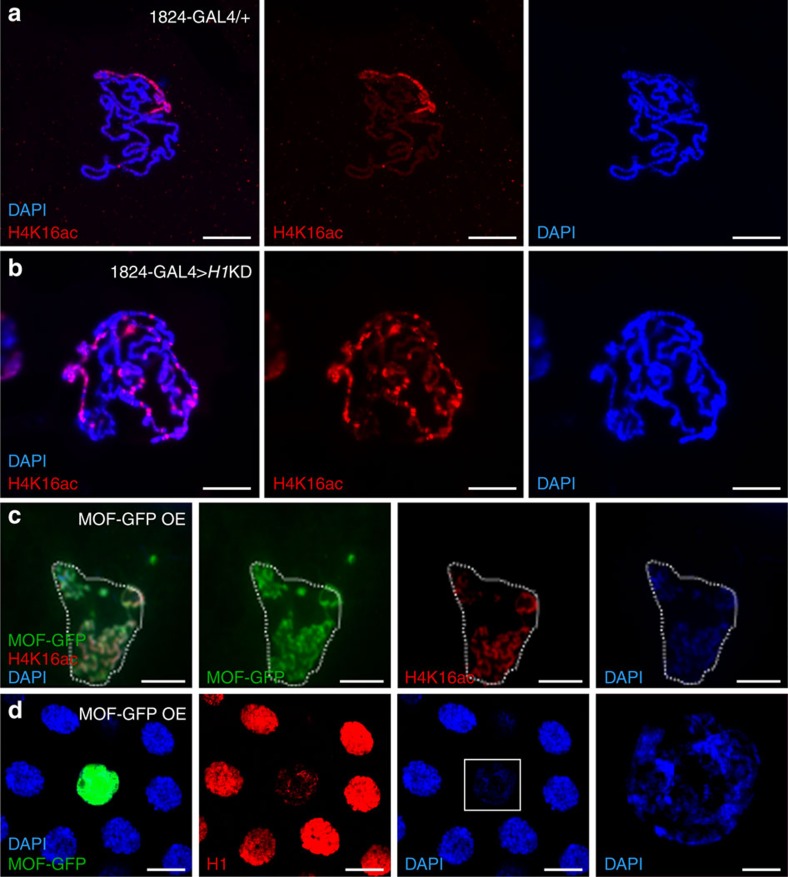
MOF antagonizes the association of H1 on chromatin. (**a**,**b**) H4K16ac is normally enriched only in the X chromosome in salivary gland cells of male third-instar larvae (**a**), but has also accumulated on autosomes in 1824-GAL4-driven *H1*KD (1824-GAL4>*H1*KD) polytene chromosomes (**b**). Note the decondensation of chromatin in **b** compared with in **a**. (**c**) Overexpression of MOF (green) causes a global increase of H4K16ac (red) on all chromosomes that co-localizes with MOF. Polytene chromosomes are from OK107-GAL4-driven UAS-MOF-GFP flies. (**d**) In MOF-overexpressing salivary gland cells (green), the association of H1 on chromatin is greatly attenuated and the decondensation of chromatin is evident, compared with that in the neighbouring control cells. The right panel is a magnified view of the rectangle area in the left panel. Note the similarities between *H1*KD (**b**) and MOF-GFP OE (**d**) phenotypes. Scale bars, 40 μm in **a** and **c**,**d**; 20 μm in **b**. DAPI, 4,6-diamidino-2-phenylindole.

As a previous study showed opposite binding trends of H1 and H4K16ac on chromatin^39^, we wondered whether H1 affects H4K16ac on the chromatin level. The H1 RNAi line that we used could also induce *H1*KD in somatic tissues (Supplementary Fig. 6a,b), we thus generated *H1*KD in the salivary gland and found that *H1*KD leads to the spread of H4K16ac from the X chromosome to autosomes (Fig. 4a,b). To further test whether H4K16 acetylation affects the H1 level on chromatin *in vivo,* we increased H4K16ac by overexpressing *mof*, the H4K16 acetyltransferase. As expected, overexpression of *mof* resulted in an increase of H4K16ac on chromosomes (Fig. 4c). Importantly, *mof* overexpression restricted H1 association on chromatin and triggered chromatin decondensation in salivary gland cells (Fig. 4d). These results show that the overexpression of *mof* behaves similarly as *H1*KD to increase H4K16ac, and prevents H1 association with the chromatin *in vivo*. We have also tested an *H4*^*K16A*^ mutant gene with the 16th lysine changed to alanine, which makes the residue incapable of being acetylated^40^. Overexpression of this transgene in the salivary gland showed that H4^K16A^ co-localizes with H1 on the polytene chromosome, in contrast to the antangonizing pattern of H1 and H4K16ac (Supplementary Fig. 7). These *in vivo* results suggest the importance of the 16th lysine in the antagonizing relationship between H1 and H4K16ac, thus further support a model of mutual repression between MOF and H1 on H4K16ac.

### Antagonism between H1 and MOF in the maintenance of GSC

**Figure 5:**
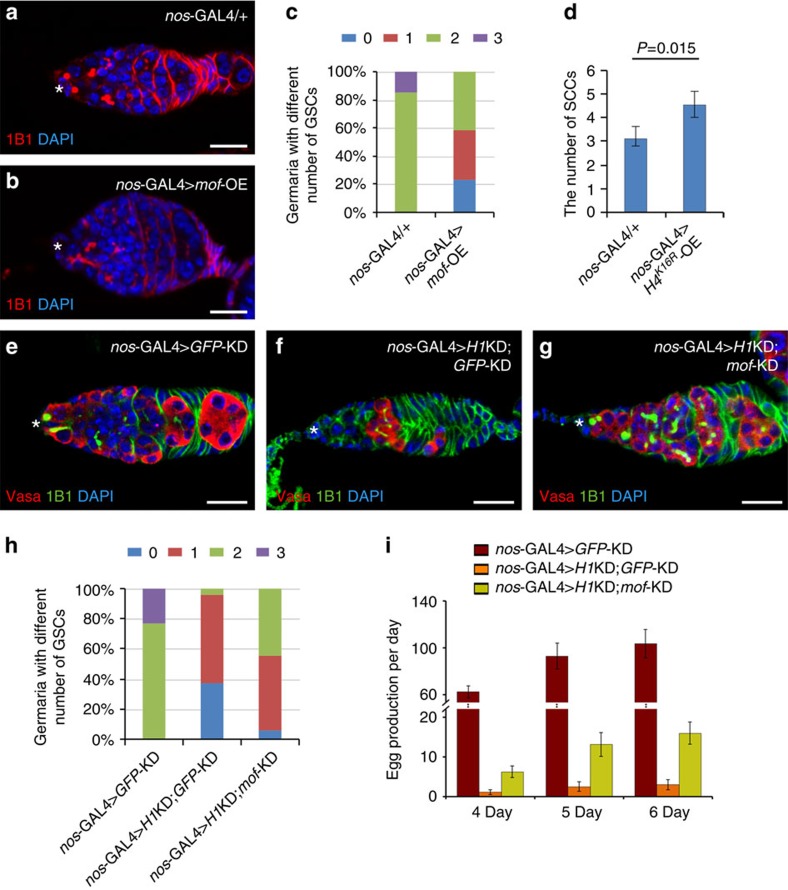
An antagonism between H1 and MOF in GSC maintenance. (**a**,**b**) Control germarium (**a**) has two GSCs that can be identified by round spectrosomes (1B1; red) and attachment to the cap cells (asterisk). A loss of GSCs can be detected by 1B1 in *mof* germline overexpression flies (**b**). Note the prematurely differentiated germline cells neighbouring the cap cells identified by the branched fusome in **b**. (**c**) Quantification of the results in **a**,**b** showing that *mof* overexpression in the germline causes GSC loss. *n*=55 and *n*=70 for control and experimental groups, respectively. (**d**) Column chart quantifying the results of the overexpression of a lysine to arginine H4 mutant (H4^K16R^) on GSC maintenance. The number of spectrosome-containing undifferentiated single-germ cells (SCCs) has significantly increased in H4^K16R^ germline overexpression germaria, compared with that in *nos*-GAL4/+ controls. *n*=21 and 24 for the two groups respectively. Data are shown as mean±s.e.m. and evaluated with Student's *t*-test. (**e**–**g**) H1 and *mof* double knockdown under the *nos* driver (**g**) results in normal numbers of GSCs similar to the control (**e**), which shows that *mof*-KD rescues the GSC loss phenotype caused by germline *H1*KD (**f**). (**h**) Quantification of the results in **e**–**g** showing that *mof*-KD can rescue the GSC loss phenotype caused by *H1*KD in the germline. *n*=81, 114 and 103 for the three groups, respectively. (**i**) Quantification of 24-h egg production rates on the 4th, 5th and 6th day after eclosion (*n*=15 for each group, mean±s.d.). Note the H1 and *mof* double knockdown significantly increases egg production compared with H1 and GFP double knockdown flies. Germaria are from 3-day-old adult flies. Scale bars, 10 μm. DAPI, 4,6-diamidino-2-phenylindole.

To determine whether elevated H4K16ac affects GSC maintenance, we used *nos*-GAL4 to overexpress *mof* to increase H4K16ac in germ cells, including GSCs. As shown in Fig. 5a–c, MOF overexpression indeed caused the GSC loss phenotype, similar to *H1*KD. Overexpression of MOF in GSCs using the mosaic assay also caused increased H4K16ac expression (Supplementary Fig. 6c) as expected. Similar to the case of MOF overexpression polytene chromosomes, where dissociation of H1 from chromatin could be observed (Fig. 4e), the level of nuclear H1 in the GSC was also reduced (Supplementary Fig. 6d). This observation is consistent with a previous study, which showed that knocking down of *mof* in the mouse embryo leads to an increased level of H1 (ref. 41). Interestingly, MOF-overexpressing GSC clones also had moderately elevated Bam levels compared with neighbouring control GSCs (Supplementary Fig. 6e), reminiscent of the similar defect in *H1*KD clones (Fig. 2e). To determine whether the lack of H4K16 acetylation affects GSC differentiation, we also used *nos*-GAL4 to overexpress H4^K16R^, a lysine to arginine mutant that mimics non-acetylated H4K16, specifically in the developing germ cells, including GSCs. Strikingly, ectopic expression of H4^K16R^ significantly increased the spectrosome-containing undifferentiated single-germ cells (Fig. 5d). These results suggest that H4K16ac is critical for the balance between GSC self-renewal and differentiation.

To test the notion that H1 and MOF control the H4K16ac levels for balancing self-renewal and differentiation of GSCs, we simultaneously depleted both H1 and MOF in germ cells, including GSCs, using *nos*-GAL4. Double H1 and MOF knockdown (KD) ovaries displayed a significant rescue of the GSC loss phenotype caused by *H1*KD (Fig. 5e–h and Supplementary Fig. 8a–c). Consequently, females with both H1 and MOF depleted in GSCs laid significantly more eggs than the H1 and GFP double KD females (Fig. 5i). These results demonstrate the antagonistic relationship between MOF and H1 in the regulation of GSC self-renewal and differentiation.

## Discussion

In this study, we used *Drosophila* ovarian GSCs as a model system to reveal that H1 and MOF antagonize each other's function to control H4K16ac levels, which are critical for GSC self-renewal and differentiation. The loss of H1 in GSCs resulted in their premature differentiation, and the derepression of *bam*, a critical regulator of GSC differentiation. In addition, our study shows a causal relationship between the knockdown of linker H1 and hyper acetylation on H4K16. Furthermore, this H4K16 hyper acetylation in the germline is specific, as many other core histone modifications do not show obvious changes, which is in contrast to the results of previous studies on somatic tissues^30^,^32^. The antagonism between H1 and MOF on chromatin *in vivo* (Figs 4 and 5), consistent with a previous *in vitro* study^42^, provides a possible explanation for the transcriptional activation of *bam* in GSCs.

The acetylation of H4K16 is a critical epigenetic modification in *Drosophila* as well as in mammals^38^,^43^. In contrast, other histone modifications, including markers of active and inactive chromatin, showed no obvious changes in ovarian germ cells, although we could not rigorously exclude the possibility that some minor changes may be beyond the detectable level of our assay or limited by the sensitivities of the antibodies. Although it is still not clear whether H4K16ac directly affects transcriptional activation in GSCs, our study has established a functional link between H1, a master transcriptional repressor, and MOF, a critical regulator of active chromatin, in GSCs. This functional interaction between these epigenetic regulators is also strongly supported by experiments showing that double depletion of H1 and MOF in GSCs can rescue the *H1*KD phenotype.

Based on our study, we propose a model to explain the functions of H1 in regulating renewal and differentiation of GSCs in *Drosophila* (Supplementary Fig. 8d). We propose that H1 supports GSC maintenance by suppressing regulators of differentiation, such as *bam* (Fig. 2). This may require at least a certain level of H1 binding on chromatin, which keeps the genes required for differentiation in an inactive state or at a ‘basal level' of transcription, without disrupting stem cell identity. We further propose that transcriptional activator MOF balances the function of H1 in GSCs by antagonizing the repressive role of H1. This model is consistent with recent studies showing that MOF resides in a complex with several activators of transcription^44^, and that the loss of MOF in mouse embryonic stem cells resulted in condensed chromatin and differentiation^45^. Interestingly, when H1 is specifically knocked down in the ECs in the germaria, which form part of the niche for the differentiation of the GSCs and cystoblasts, we have observed an increase of GSC-like, spectrosome-containing cells in the germaria, which resembles an ovarian tumour. Strikingly, this phenotype can also be suppressed by the concomitant knocking down of *mof*, which also supports our model (Supplementary Fig. 9). Taken together, our results support that linker H1 is a regulator with a critical function in the regulation of GSC self-renewal, and MOF antagonizes this function through H4K16 acetylation. It will be interesting to test whether this mechanism is conserved in mammals, and whether this novel mechanism can explain how dysregulated H1 contributes ovarian tumorigenesis in humans in the future.

## Methods

### *Drosophila* husbandry and genotype information

The following fly stocks were used in this study: *UAS-H1KD* RNAi lines (TH00868.N, TH00825.N, and TH00826.N), a *UAS-mof* RNAi line (TH00870.N), *UAS-dBigH1* RNAi lines (TH11322.N and TH11323.N), *UAS-GFP* RNAi line (TH00782.N), *UAS-GFP* (TH10512.N), *P[nosP-GAL4::vp16]*^46^, *y, hs-FLP; nos<STOP<GAL4::VP16, UASp-GFP/CyO*^29^, *P[bamP-GFP]*^22^, *Dad-LacZ*^35^, *bam*^*Δ86*^ (ref. 47), *UAS-p35* (ref. 48), *1824-GAL4* (*AB1-GAL4*; Bloomington #1824), *OK107-GAL4* (w*;;;P{GawB}ey^OK107^, a gift from Dr Yi Zhong) and *c587-Gal4* (Bloomington #25421).

Sense Oligos of the transgenic RNAi lines are:

TH00782.N: 5′-CCCGAAGGTTATGTACAGGAA-3′

TH00825.N: 5′-AAGCAAGAAGGTAGCCTCTAA-3′

TH00826.N: 5′- TAGCGAAAGCGTCAAAGGCAA-3′

TH00868.N: 5′-ACCAGCGACAGTTGAGAAGAA-3′

TH00870.N: 5′-CTCGACCTCAGCGGTGTCCAA-3′

TH11322.N: 5′-CGGCGAAGTGGTGATGGTTAA-3′

TH11323.N: 5′-ATGGTTAAGCGATCCTTTAAA-3′.

All *Drosophila* stocks were maintained at 25 °C with 60% humidity on standard cornmeal/sugar/agar media unless otherwise specified.

### Transgene constructs and production of transgenic flies

H1, MOF::GFP fusion protein and H4^K16R^ transgene were constructed using a previously described method and a ValiumP vector^27^. Transgenes expressing H1 and MOF::GFP fusion proteins were constructed as follows: the H1-coding sequence was amplified (forward primer: 5′-GGTCTAGAATGGCCATGTCTGATTCTGCAGTTGCA-3′; reverse primer: 5′-AATCTAGATTACTTTTTGGCAGCCGTAGTC-3′). The H1 PCR product was digested with *Xba*I and cloned into a ValiumP vector^27^. The *mof*-coding sequence was amplified (forward primer: 5′-AACCTAGGATGGCCATGTCTGAAGCGGAGCTGGA-3′; reverse primer: 5′-AAGAATTCGCCGGAATTTCCCGGAGCT-3′). The PCR product was digested with *Avr*II/*Eco*RI and cloned into the ValiumP-GFP vector.

The full cDNA of histone H4 was amplified (forward primer: 5′-GGTCTAGAATGGGAATGACTGGTCGTGGTAAAGG-3′; reverse primer: 5′-AAGGATCCTTAACCGCCAAATCCGTAGAG-3′). The PCR product was digested with *Xba*I/*Eco*RI and cloned into the ValiumP-GFP vector. The *H4*^*K16R*^ gene was then generated by converting the AAG codon into AGG using the AccuPrime Pfx DNA Polymerase Kit (Invitrogen) according to the manufacturer's instructions. Lysine 16 was replaced with arginine (H4^K16R^) in H4-GFP.

Transgenic fly lines were produced by injecting the constructs into *y sc v nanos-integrase; attP2* or *y sc v nanos-integrase; attP40* stocks following the standard procedure^27^.

For the overexpression of MOF and H4^K16A^ in salivary glands, the MOF::GFP- and H4::GFP-coding sequences were subcloned into pUAST vectors, in which MOF::GFP and H4::GFP were flanked by hsp70 basal promoter and the SV40 polyA tail. The *H4*^*K16A*^ gene was then generated by converting the 16th codon AAG (Lysin) into GCG (Alanine). The plasmids were then injected into *w*^*1118*^ embryos following the standard procedure^49^.

### FLP-out and OK107-induced clonal analysis

Clones of RNAi cells in the ovary were generated by FLP/FRT-mediated recombination. To generate GSC clones, *y, hs-FLP; nos<STOP<GAL4::VP16, UASp-GFP/CyO* or *y, hs-FLP; nos<STOP<GAL4::VP16, UASp-GFP/CyO; Dad-lacZ/TM6B* was crossed with the *UAS-H1KD RNAi line* (TH00868.N). Adult females of the appropriate genotypes were heat-shocked at 37 °C for 45 min 2 days after eclosion. The females were then transferred to fresh food at 29 °C, and the ovaries were dissected at days 2, 7, 14 and 21 after heat-shock treatment for antibody staining.

Mosaic salivary glands with RNAi cells were generated using the *OK107-GAL4* driver. *OK107-GAL4* is mostly expressed in the mushroom bodies, but is also expressed in a random manner in a few salivary gland cells^40^. Thus, *OK107* was applied to generate random RNAi and overexpression mosaics to study salivary gland cells and polytene chromosomes.

### Immunostaining of ovaries

Immunostaining of ovaries followed previously described protocols^29^. Ovaries were dissected in cold PBS, fixed in PBS with 4% formaldehyde for 15 min and then washed with PBT (PBS and 0.1% Triton X-100) five times for 15 min each. The ovaries were first incubated in 0.5% goat serum diluted in PBT for 1 h and then with the appropriate primary antibodies diluted in PBS at 4 °C overnight. The samples were then washed with PBT five times for 15 min each, incubated with the appropriate secondary antibodies at 25 °C for 2 h, then washed with PBT five times for 15 min each. After the last wash, the stained ovaries were mounted in Fluoromount mounting media (Sigma, F4680). Images were obtained with an inverted Zeiss LSM780 fitted with a ultraviolet laser. The NIS Elements BR programme was used for measurements.

The following primary antibodies were used: mouse monoclonal anti-Hts antibody 1B1 (Developmental Studies Hybridoma Bank (DSHB), 1:100), mouse monoclonal anti-LaminC antibody LC28.26 (DSHB, 1:100), rabbit polyclonal anti-Vasa (Santa Cruz Biotechnology, sc30210, 1:200), rabbit polyclonal anti-H1 (anti-H1C; Fang-Lin Sun)^28^, rabbit polyclonal anti-GFP (Abcam, ab290, 1:1,000), mouse monoclonal anti-GFP (Roche, 1814460,1:50), anti-pMad (Cell Signaling, 9516, 1:50), anti-beta-Galactosidase (Cappel, 55976, 1:1,000), rabbit polyclonal anti-cleaved Caspase-3 (Cell Signaling, 9661, 1:200), rabbit polyclonal anti-dBigH1 (a gift from Dr Fernando Azorín, 1:40), rabbit polyclonal anti-H4K16ac (Millipore, 07-329, 1:200), rabbit polyclonal anti-H4K12ac (Abcam, ab46983, 1:400), rabbit polyclonal anti-H4K8ac (Cell Signaling, 2594, 1:200), rabbit polyclonal anti-H4K5ac (Millipore, 07-327, 1:100), rabbit polyclonal anti-H3K4me2 (Millipore, 07-030, 1:100), rabbit polyclonal anti-H3K27me3 (Cell Signaling, 9733, 1:200), and rabbit polyclonal anti-H3K9me2 (Millipore, 07-212, 1:200). Various secondary antibodies (Jackson ImmunoResearch Laboratories) conjugated with FITC or TRITC were used at 1:200.

To quantify the immunostaining results of the mosaic studies, areas of interest (identified by markers including GFP, LaminC and 4,6-diamidino-2-phenylindole) were selected using the freeform tool in ImageJ and measured to get the Mean Density readings. Then the light intensities of RNAi or overexpression GSC clones were directly compared with that of the neighbouring control GSCs (no GFP expression). Average light intensity results from five independent pictures were recorded. Error bars denote standard deviation.

### Reverse transcription–PCR and qRT–PCR

Total RNA was isolated from *Drosophila* ovaries 3 day after eclosion using the AxyPrep Multisource Total RNA MiniPrep Kit (Axygen). A total of 1 μg RNA was used to create cDNA, using the GoldScript cDNA Kit (Invitrogen) according to the manufacturer's protocol, followed by PCR to amplify the target sequence. The actin 5C gene (*act5C*) served as an internal control.

qRT–PCR was performed using SYBR Premix Ex Taq (TAKARA) and analysed with the iQ5 real-time PCR detection system (Bio-Rad). Results were normalized against *rp49* expression.

Primer sequences for reverse transcription–PCR experiments are listed below:

act5c-F: 5′-ATACTCCTCCCGACACAAAGC-3′

act5c-R: 5′-CAGGTAGTCGGTCAAATCGC-3′

H1-F: 5′-ggTCTAGAATGGCCATGTCTGATTCTGCAGTTGCA-3′

H1-R: 5′-aactcgagTTACTTTTTGGCAGCCGTAGTC-3′

mof-F: 5′-CGATTGAGGAGGAGCATGAG-3′

mof-R: 5′-CAATTCAACTGGACCTGGTG-3′

Primer sequences for qRT–PCR experiments are listed below:

rp49-qF: 5′-ATCGGTTACGGATCGAACAAGC-3′

rp49-qR: 5′-GTAAACGCGGTTCTGCATGAGC-3′

H1-set-1-qF: 5′-CAAAGCTAAGAAGGCTGTGG-3′

H1-set-1-qR: 5′-GGCTTCGACTTTATGATTCCAG-3′

H1-set-2-qF: 5′-TAAGGGAAAGGGTGCATCTG-3′

H1-set-2-qR: 5′-CTTAGAGGCTACCTTCTTGC-3′

bam-qF: 5′-CGAGGATACGAACGAAGATGG-3′

bam-qR: 5′-GAATTCGAGGAGTGGTGCAG-3′

dBigH1-qF: 5′-TGAAGGAAAAGAAGGCCTCC-3′

dBigH1-qR: 5′-TAGATGCTGGCGGATTATCC-3′.

### Western blot

Extracts were prepared from 3-day-old adult ovaries. For each genotype, 80 ovaries were collected and lysed in NP-40/300 mM NaCl buffer (1% NP-40, 300 mM NaCl, 50 mM Tris at pH 7.8, Roche protease inhibitor). The protein concentration of the supernatant was measured by using Bio-Rad Protein Assay Reagent (500-0006). For SDS–polyacrylamide gel electrophoresis, 10 μg per lane were loaded. After electrophoresis, proteins were transferred from the gel onto polyvinylidene difluoride membrane (Bio-Rad), then hybridized with primary antibodies at the dilutions indicated: anti-H1C (1:2,000), anti-H3 (Abcam, ab1791, 1:5,000). The secondary antibodies were peroxi-dase-conjugated affinipure goat anti-rabbit IgG (H+L) (1:5,000). The ECL detection system (Thermo) was used to detect signals on the blots.

### Egg-laying assay

To measure egg-laying rates, virgin females were collected within 6 h of eclosion, and two (for control vials) or seven (for experimental vials) females were mated to five *w*^*1118*^ males. Females were transferred to new vials containing fresh food with several grains of yeast every day for the duration of the experiment. Egg production by individual females was scored by counting the number of eggs laid in successive 24-h periods and dividing by the number of females in each vial. Approximately 15 vials were scored for each group.

### Polytene chromosome staining

Immunostaining of third-instar larval polytene chromosomes was performed as previously described^50^. Chromosomes were incubated with rabbit polyclonal anti-H1C (1:500), rabbit polyclonal anti-acetylated histone H4K16 (anti-H4K16ac) or mouse monoclonal anti-GFP (MBL M048-3, 1:500) at room temperature for 1 h. After washing with PBS, chromosomes were incubated with a secondary goat anti-mouse antibody conjugated to FITC (1:100) and a goat anti-rabbit antibody conjugated to Texas red (1:100; Jackson ImmunoResearch Laboratories) at room temperature for 2 h. After one PBS wash, the slides were incubated with 4,6-diamidino-2-phenylindole to stain DNA (Sigma, 1:1,000). The immunostained slides were mounted, and the images were taken using a Leica DMI 4000 B inverted microscope (Leica; × 40 model) and processed using Adobe Photoshop software.

## Additional information

**How to cite this article:** Sun, J. *et al.* Histone H1-mediated epigenetic regulation controls germline stem cell self-renewal by modulatingH4K16 acetylation. *Nat. Commun.* 6:8856 doi: 10.1038/ncomms9856 (2015).

## Supplementary Material

Supplementary Information Supplementary Figures 1-9
