# Supplementary material for: Histone H1-mediated epigenetic regulation controls germline stem cell self-renewal by modulating H4K16 acetylation
Source: Nat Commun. 2015 Nov 19;6:8856. doi: 10.1038/ncomms9856 (PMC4673494; doi:10.1038/ncomms9856)
Supplement: Supplementary Information — Supplementary Figures 1-9 [file ncomms9856-s1.pdf]

Supplementary Information

Histone H1 Mediated Epigenetic Regulation Controls Germline Stem Cell Self-Renewal by Modulating H4K16 Acetylation

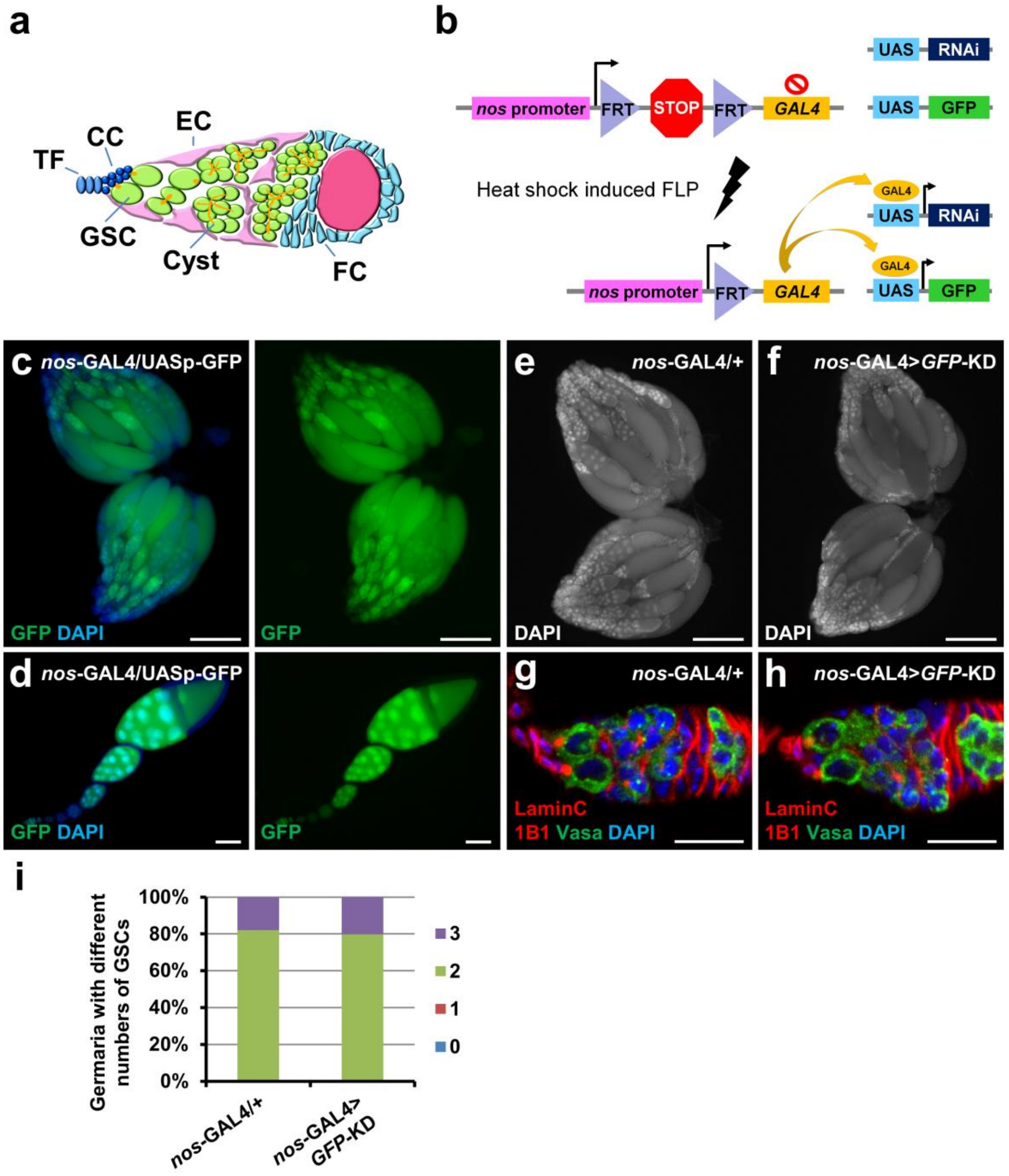

Supplementary Figure 1 Some of the tools used in this study. (**a**) Schematic showing a germarium. Anterior is to the left. There are two or three germline stem cells (GSCs; green) at the anterior tip of the germarium, attached to a niche that includes the cap cells (CC; blue) and escort cells (EC; pink). The germline lineage is derived from the GSCs (green). Terminal filament (TF) cells, light blue. Follicle cells (FC), cyan. Spectrosomes, organelles of GSCs and cystoblast, and fusomes, branched organelles of the differentiated cysts, are shown in orange. (**b**) Cartoon diagram showing the FLP-out RNAi clonal system. Before heat shock, transcription stops at the transcription termination between the two FRTs, and these cells do not express GFP. After heat shock-induced FLP expression, some cells lose the DNA sequence between the FRTs, and GAL4 expression in these cells trigger both GFP expression and shRNA expression to knockdown the target mRNA. (**c**-**d**) *nos*-GAL4 drives expression of GFP in the germline (**d**) of whole ovaries (**c**). *nos*-GAL4 is expressed in the germline in all stages of oogenesis, but at weaker levels in stage 2-4 egg chambers. (**e**-**h**) Both heterozygous *nos*-GAL4 (*nos*-GAL4/+) (**e** and **g**) and *nos*-GAL4 driven GFP RNAi (*nos*-GAL4>*GFP*-KD) (**f** and **h**) flies do not show any phenotypes in the ovaries. (**i**) Column chart showing the percentages of germaria with different numbers of GSCs from *nos*-GAL4/+ and *nos*-GAL4>*GFP*-KD controls. Both control lines show similar numbers of GSCs in the germaria. *n* = 106 and 114 for the two groups, respectively. Scale bars: 100 µm in (**c**-**f**); 10 µm in (**g**-**h**).

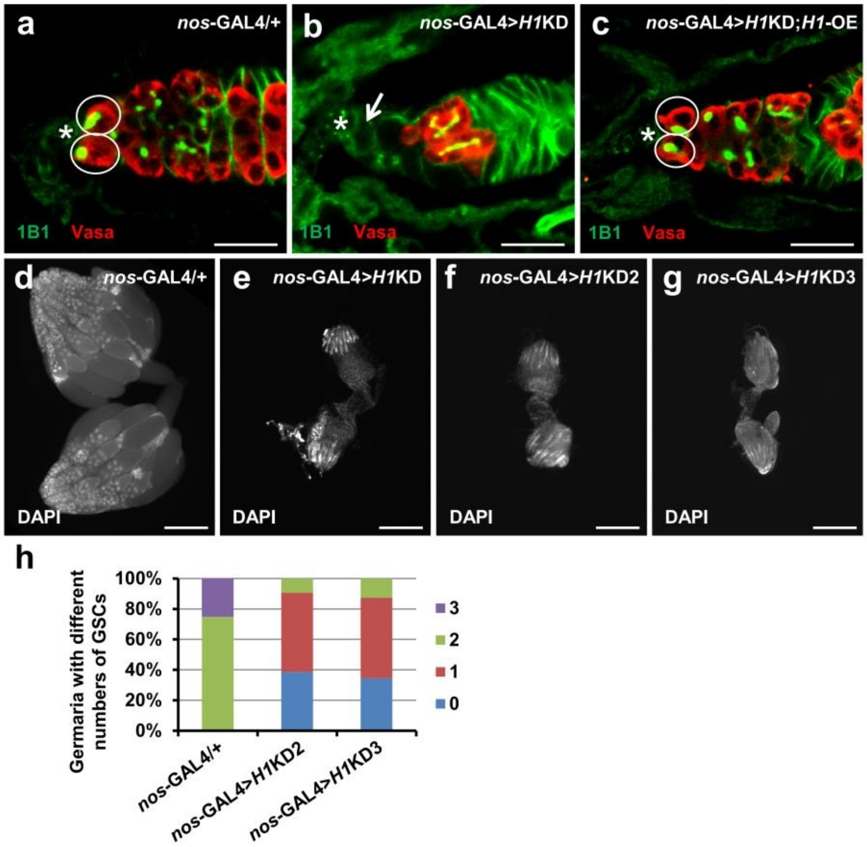

Supplementary Figure 2 Confirming the H1 phenotypes in the germline. (**a**-**c**) Germaria are from 3-day-old adult flies, and are stained with 1B1 (green) to reveal spectrosomes/fusomes and anti-Vasa (red) to show the germ cells. Germaria from controls (**a**) have two GSCs (circles), while those from *nos*-GAL4>*H1*KD (**b**) have no GSC (arrow). (**c**) *H1*-OE rescues the loss of GSCs. (**d**-**g**) *H1*KD2 (**f**) and *H1*KD3 (**g**) result in small ovaries when driven by *nos*-GAL4, similar to the *H1*KD germline defects (**e**). Images show whole ovaries from 3-day-old adults flies stained with DAPI. (**h**) Column chart showing the percentages of germaria with different numbers of GSCs from control, *nos*-GAL4>*H1*KD2, and *nos*-GAL4>*H1*KD3 flies. Both H1 RNAi lines show a decrease of GSC numbers in the germaria when driven by *nos*-GAL4, at a level similar to that of *nos*-GAL4>*H1*KD (Fig. 1d). *n* = 103, 96, and 87 for the three groups, respectively. Scale bars: 10 µm in (a-c); 100 µm in (d-g).

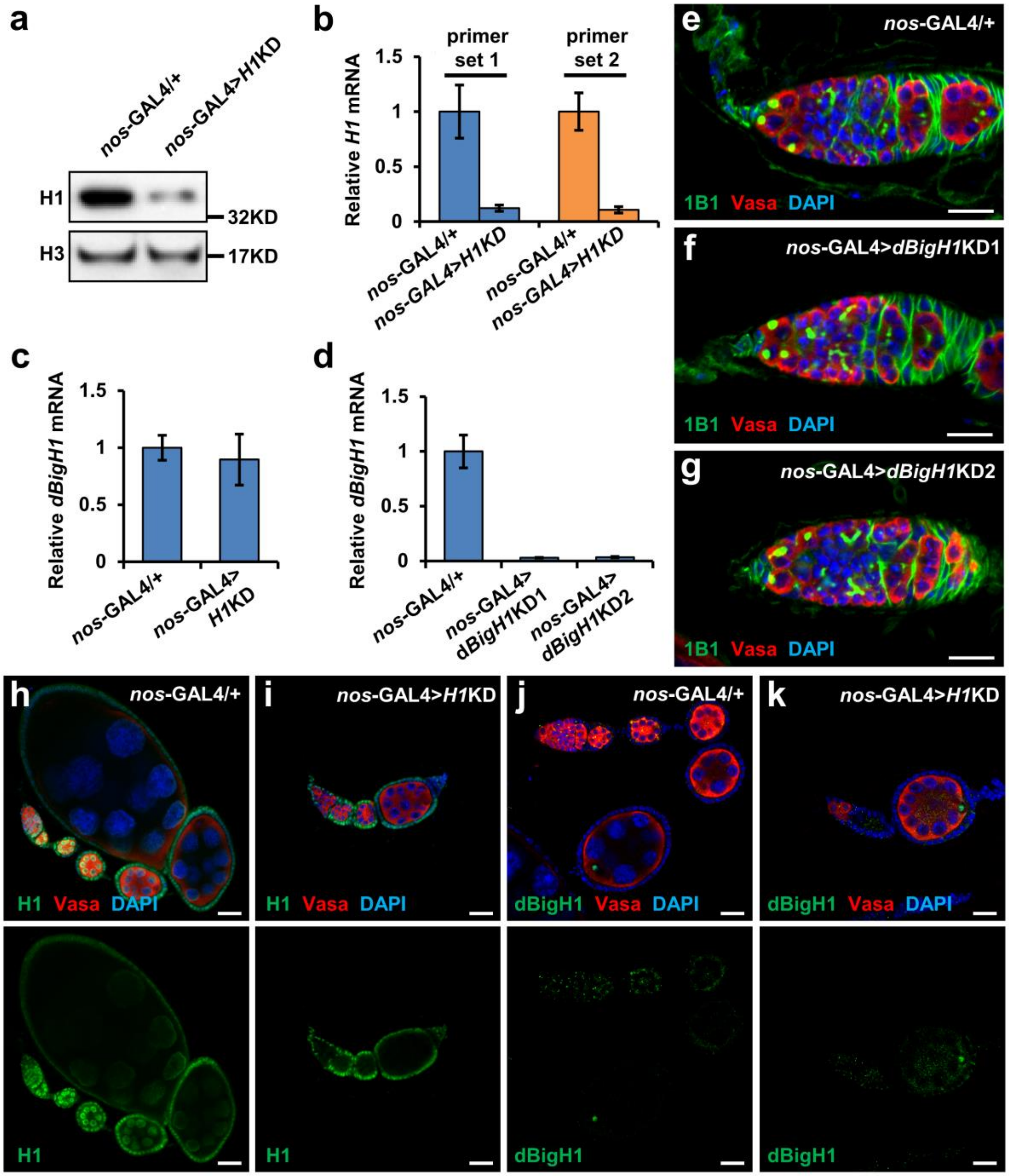
a
nos-GAL4/+
nos-GAL4>H1KD
H1
H3
32KD
17KD
b
primer set 1
primer set 2
Relative H1 mRNA
1.5
1
0.5
0
nos-GAL4/+
nos-GAL4>H1KD
nos-GAL4/+
nos-GAL4>H1KD
c
Relative dBigH1 mRNA
1.5
1
0.5
0
nos-GAL4/+
nos-GAL4> H1KD
d
Relative dBigH1 mRNA
1.5
1
0.5
0
nos-GAL4/+
nos-GAL4> dBigH1KD1
nos-GAL4> dBigH1KD2
e
nos-GAL4/+
1B1 Vasa DAPI
f
nos-GAL4>dBigH1KD1
1B1 Vasa DAPI
g
nos-GAL4>dBigH1KD2
1B1 Vasa DAPI
h
nos-GAL4/+
H1 Vasa DAPI
H1
i
nos-GAL4>H1KD
H1 Vasa DAPI
H1
j
nos-GAL4/+
dBigH1 Vasa DAPI
dBigH1
k
nos-GAL4>H1KD
dBigH1 Vasa DAPI
dBigH1

Supplementary Figure 3 dBigH1 is not involved in H1's roles in GSC maintenance. (**a**) Western blot shows that H1 protein dramatically decreases in *H1*KD ovaries. (**b**-**c**) qRT-PCR results from the same ovary samples show that the transcription of H1 in *nos*-GAL4>*H1*KD flies is reduced compared with that in the *nos*-GAL4/+ control (**b**), while dBigH1 level remains unchanged (**c**). Two different sets of H1 primers were tested in (**b**). *n*=3, mean ±s.d. (**d**) qRT-PCR results show that dBigH1 levels have been efficiently reduced in the dBigH1 germline knockdown ovaries (*nos*-GAL4>*dBigH1*KD1 and *nos*-GAL4>*dBigH1*KD2) compared with that in the *nos*-GAL4/+ control. *n*=3, mean ±s.d. (**e**-**g**) Two independent dBigH1 knockdown lines show no GSC phenotypes. Germaria are from 3-day-old adult flies, and are stained with 1B1 (green) to reveal spectrosomes/fusomes, anti-Vasa (red) to show the germ cells, and DAPI (blue) to show the nuclei. (**h**) H1 (green) is expressed both in the somatic cells and the germline cells during oogenesis, but at weaker levels as egg chambers mature. H1 is not expressed in the oocytes and is at a reduced level in the nurse cells neighbouring the oocyte. (**i**) H1 decreases to undetectable levels in germline cells that are marked by anti-vasa staining, compared with those in the controls (h). (**j**-**k**) H1 knockdown in the germline does not affect the expression of dBigH1 (green). Germaria are from 3-day-old adult flies. Note the strong expression of dBigH1 in the oocyte nuclei in (**j**) and (**k**). Scale bars: 10 μm in (**e**-**g**); 20 μm in (**h**-**k**).

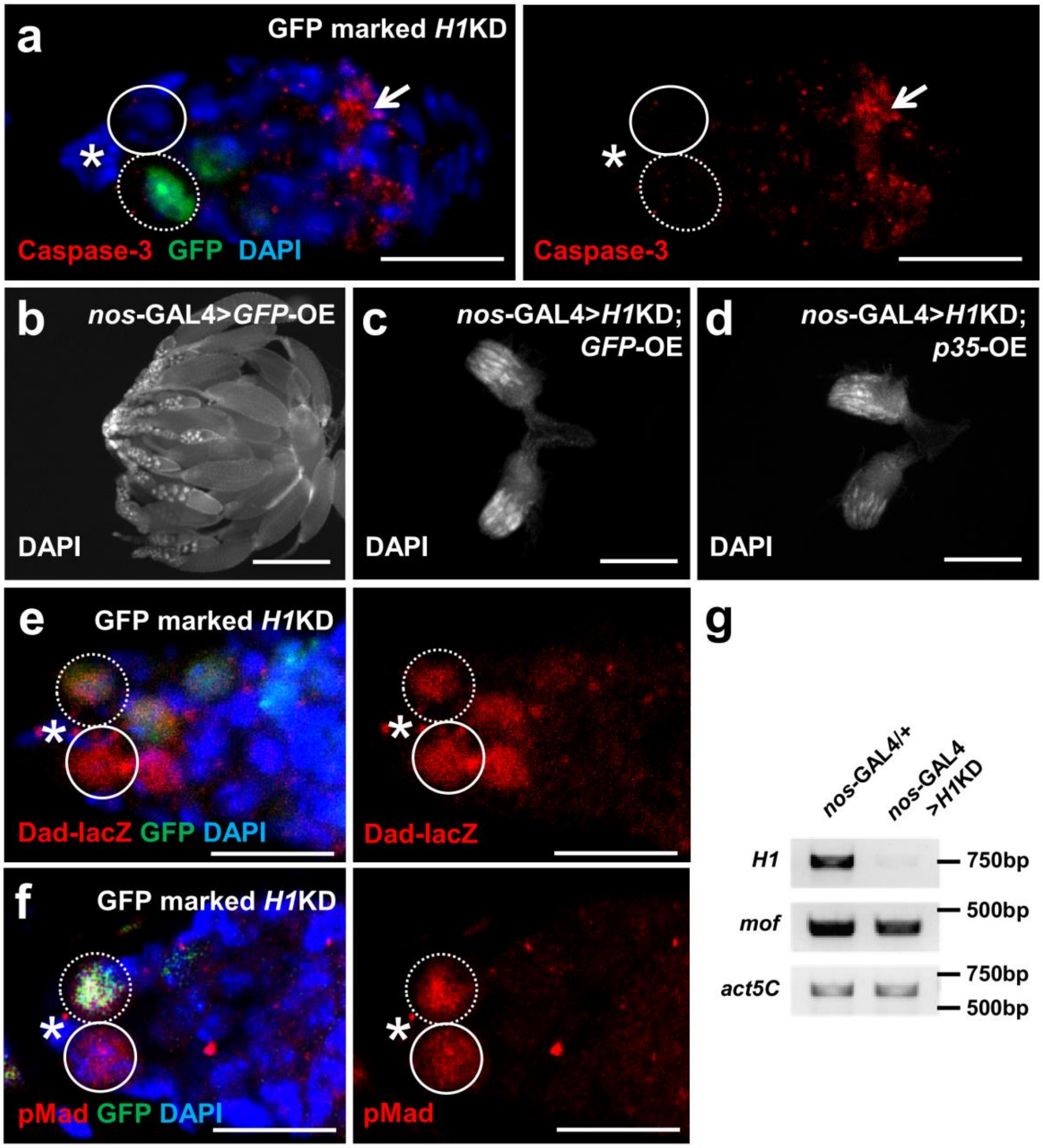

a
GFP marked H1KD
Caspase-3 GFP DAPI
Caspase-3
b
nos-GAL4>GFP-OE
DAPI
c
nos-GAL4>H1KD; GFP-OE
DAPI
d
nos-GAL4>H1KD; p35-OE
DAPI
e
GFP marked H1KD
Dad-lacZ GFP DAPI
Dad-lacZ
f
GFP marked H1KD
pMad GFP DAPI
pMad
g
nos-GAL4/+
nos-GAL4 >H1KD
H1
mof
act5C
750bp
500bp
750bp
500bp

Supplementary Figure 4 Germline H1 knockdown phenotypes do not involve Dpp signalling, or caspase-dependent apoptosis. (**a**) The expression of Caspase-3 (red) remains undetectable in the GFP positive *H1*KD GSCs (broken circle), compared with that in the GFP negative control GSCs (circles). Germaria are from flies bearing *H1*KD GSC clones (green) 7 days ACI. Arrow points to Caspase-3 active cells, indicating that the antibody against cleaved Caspase-3 is functional. 387 germaria examined. (**b**-**d**) p35 overexpression (**d**) does not suppress the small ovary phenotype caused by H1 depletion (**c**). Images show whole ovaries from 3-day-old adult flies stained with DAPI. Ovaries are from GFP overexpression control (*nos*-GAL4>*GFP*-OE) (**b**), *nos*-Gal4 driven *H1*KD and GFP overexpression (*nos*-GAL4>*H1*KD; *GFP*-OE) (**c**), and *nos*-Gal4 driven UAS-*H1*KD and p35 overexpression (*nos*-GAL4>*H1*KD; *p35*-OE) (**d**) flies. (**e**-**f**) The expression of Dad-lacZ (red in **e**) and pMad (red in **f**) in the GFP positive *H1*KD GSCS (broken circle) remain at similar intensities as those in the GFP negative control GSCs (circles). Confocal images show germaria from flies bearing *H1*KD GSC clones 7 days ACI. (**g**) RT-PCR results comparing gene expression levels between in *nos*-GAL4/+ control ovaries and in *nos*-GAL4 driven UAS-*H1*KD (*nos*-GAL4>*H1*KD) ovaries. Levels of *act5C* expression are used as the loading control. No obvious differences can be detected on *mof* expression. Decreased H1 expression can be noticed. Scale bars: 10 µm in (**a**) and (**e**-**f**); 100 µm in (**b**-**d**).

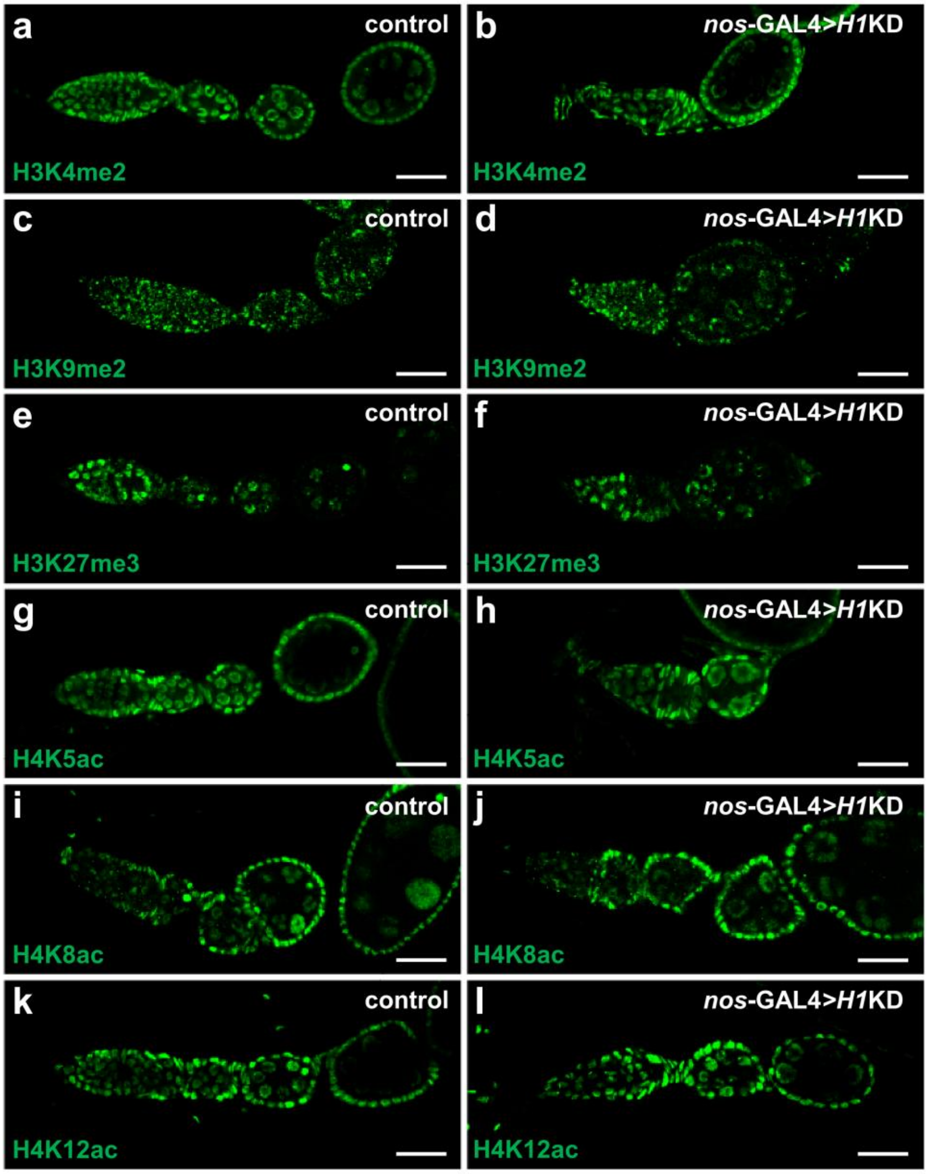

Supplementary Figure 5 *H1*KD does not change the expression of most histone modifications tested. (**a**-**l**) The expression levels of the examined histone modifications (green) are indistinguishable between in the *nos*-GAL4/+ control (**a**, **c**, **e**, **g**, **i**, **k**) and *nos*-GAL4>*H1*KD (**b**, **d**, **f**, **h**, **j**, **l**) flies 3 days after eclosion. Germaria are stained to examine the expression levels of H3K4me2 (**a** and **b**), H3K9me2 (**c** and **d**), H3K27me3 (**e** and **f**), H4K5ac (**g** and **h**), H4K8ac (**i** and **j**), and H4K12ac (**k** and **l**), respectively. Scale bars, 25 μm.

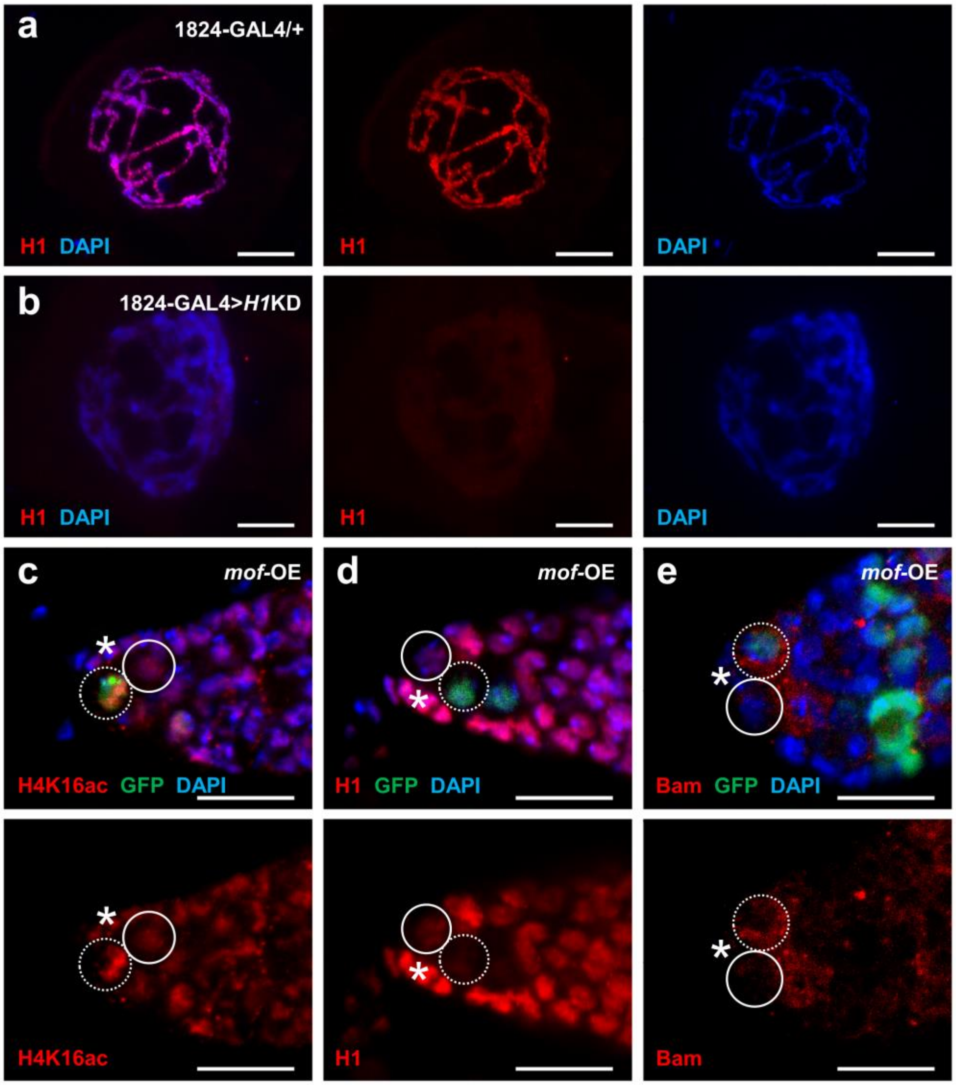

Supplementary Figure 6 Confirming the antagonism between H1 and MOF. (**a**- **b**) Knockdown of H1 (red) causes more relaxed chromatins. Polytene chromosomes are from 1824-GAL4/+ control (**a**) and 1824-GAL4-driven UAS-*H1*KD flies (1824-GAL4>*H1*KD) (**b**). (**c**-**e**) Overexpression of MOF (green) causes an increase of H4K16ac (**c**), a decrease of H1 expression (**d**), and an increase of Bam (**e**) in the GSCs of ovaries. Mosaic ovaries with MOF overexpression germline clones (GFP positive; green) are generated by heat shock and are observed 5 days after clone induction in (**c**-**e**). Scale bars: 40 μm in (**a**-**b**); 10 μm in (**c**-**e**).

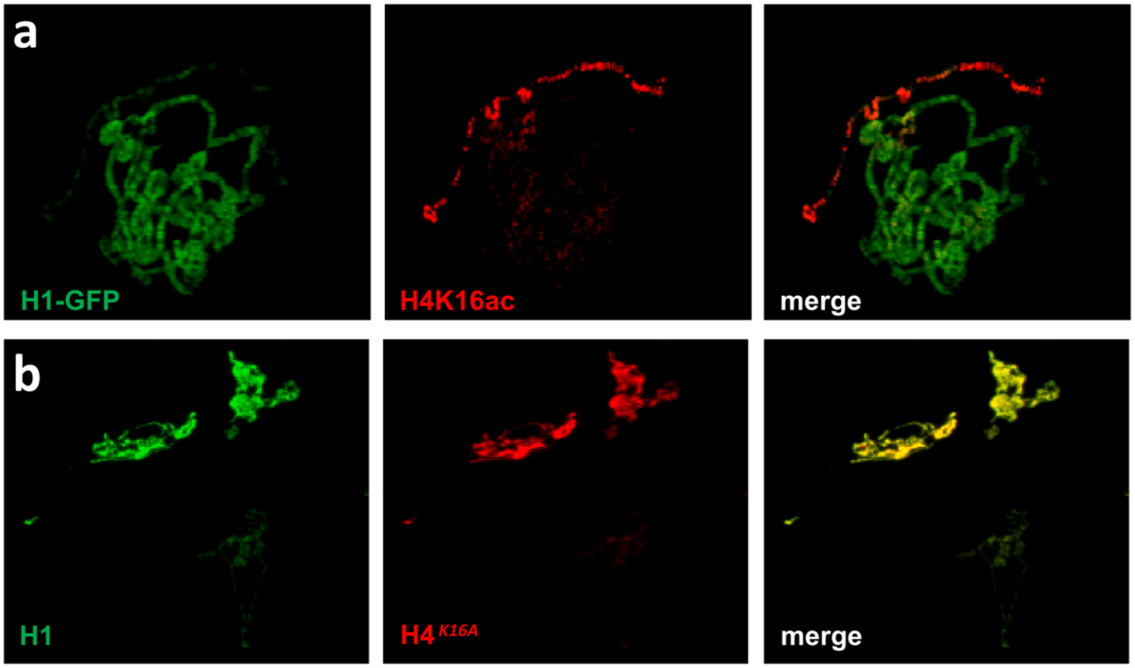

Supplementary Figure 7. Un-acetylatable H4^{K16A} mutant colocalizes with H1. (a) Acetylated H4K16 (H4K16ac; red) on polytene chromosomes excludes H1 (H1-GFP; green). H4K16ac is normally enriched only in the X chromosome in salivary gland cells of male 3rd-instar larvae. (b) The expression of an un-acetylatable H4^{K16A} mutant with the 16th lysine changed to alanine (red) colocalizes with H1 (green) on polytene chromosomes. H1-GFP and the H4^{K16A} transgenes are driven by 1824-GAL4. Polytene chromosomes are from 3rd instar larvae.

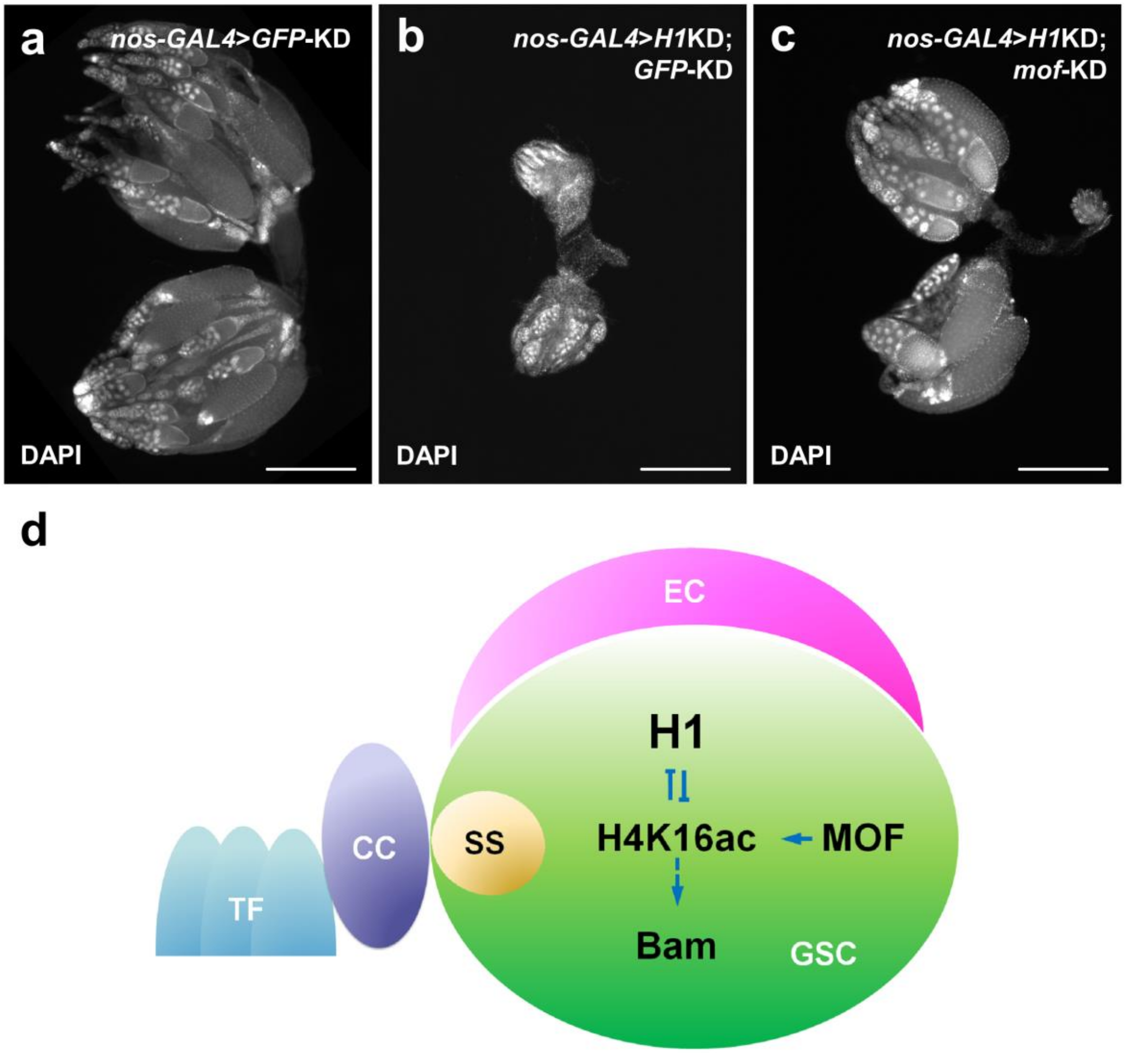
a
nos-GAL4>GFP-KD
DAPI
b
nos-GAL4>H1KD;
GFP-KD
DAPI
c
nos-GAL4>H1KD;
mof-KD
DAPI
d
EC
H1
CC
SS
H4K16ac
MOF
TF
Bam
GSC

Supplementary Figure 8 A balance between H1 and MOF maintain the proper control of GSC self-renewal and differentiation. (**a**-**c**) H1 and *mof* double knockdown under the *nos*-GAL4 driver (**c**) results in ovaries of sizes comparable to those of the *nos*-GAL4>*GFP*-KD controls (**a**), a significant rescue from the rudimentary ovaries of the *nos*-GAL4>*H1*KD; *GFP*-KD flies (**b**). Images show whole ovaries stained with DAPI. Ovaries are dissected from 3-day-old adult flies. Scale bars: 100 µm. (**d**) A cartoon model showing half of the anterior tip of a germarium to illustrate H1-mediated regulation of GSC maintenance. In this model, a balance between H1 and MOF is required for the proper control of GSC self-renewal and differentiation. This balance ensures the suppression of *bam* in GSCs to maintain the identity of GSCs before differentiation. MOF antagonizes H1 through H4K16ac, which attenuates H1 association on chromatin. Knocking-down of H1 in GSCs tips the balance and causes premature differentiation of GSCs. An H1 and MOF double knockdown resets the balance and thus suppresses the phenotypes. GSC, germline stem cell (green); EC, escort cell (magenta); CC, cap cell (purple); TF, terminal filament cell (cyan); SS, spectrosome (yellow).

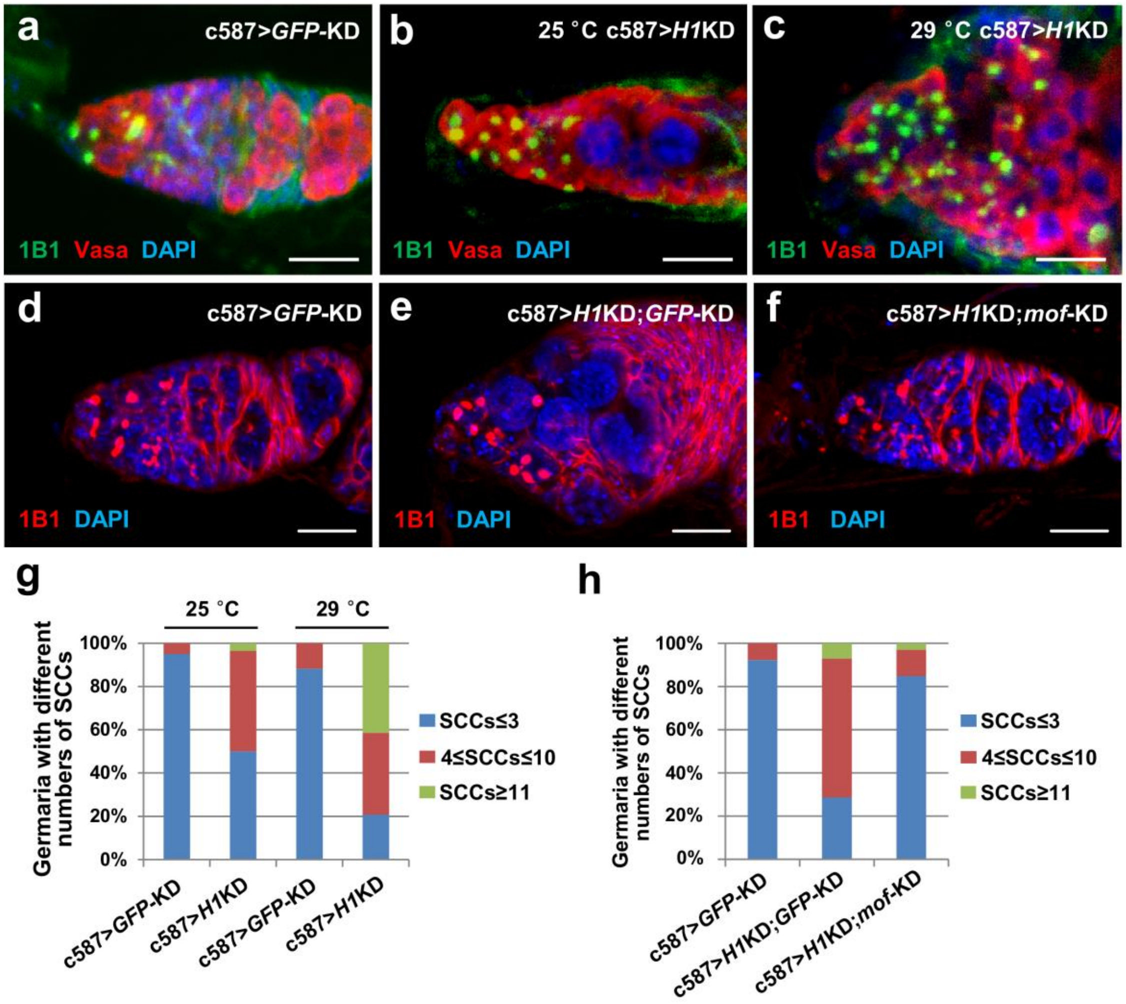
a
c587>GFP-KD
1B1 Vasa DAPI
b
25 °C c587>H1KD
1B1 Vasa DAPI
c
29 °C c587>H1KD
1B1 Vasa DAPI
d
c587>GFP-KD
1B1 DAPI
e
c587>H1KD;GFP-KD
1B1 DAPI
f
c587>H1KD;mof-KD
1B1 DAPI
g
25 °C
29 °C
Germaria with different numbers of SCCs
100%
80%
60%
40%
20%
0%
c587>GFP-KD
c587>H1KD
c587>GFP-KD
c587>H1KD
SCCs≤3
4≤SCCs≤10
SCCs≥11
h
Germaria with different numbers of SCCs
100%
80%
60%
40%
20%
0%
c587>GFP-KD
c587>H1KD;GFP-KD
c587>H1KD;mof-KD
SCCs≤3
4≤SCCs≤10
SCCs≥11

Supplementary Figure 9. H1 is required non-autonomously to suppress GSC differentiation and GSC-like tumors. (a-c) Confocal images of germaria from *c587*-GAL4/+ controls raised at 25°C (a), *c587*-Gal4-driven H1 knockdown (c587>H1KD) raised at 25°C (b), and c587>H1KD raised at 29°C (c). Germaria were stained with 1B1 (green) to demonstrate spectrosomes/fusomes and anti-Vasa (red) to show the germline. Note the GSC-like spectrosome-containing cells (SCCs) have caused a tumor-like phenotype in (b) and (c). (d-f) Confocal images of germaria from *c587*-Gal4-driven GFP knockdown control (c587>GFP-KD) (d), *c587*-Gal4-driven H1 and GFP knockdown (c587>H1KD;GFP-KD) (e), and c587-Gal4-driven knockdown of both H1 and mof (c587>H1KD;mof-KD) (f) flies, stained with 1B1 (red), and DAPI (blue). Note that the GSC-like tumor phenotype caused by H1KD in the escort cells (e) was rescued by mof-KD (f). (g) Column chart quantifying the changes in the percentages of round SCCs in the germaria of control and c587>H1KD flies in (a-c). An increase in SCCs was observed in c587>H1KD flies at 25°C and 29°C compared with the c587>GFP-KD controls. *n* = 59, 57, 51 and 48 for the four groups, respectively. (h) Column chart quantifying the results shown in (d-f). The Y-axis shows the percentage of germaria with different numbers of SCCs. The percentage with abnormal numbers of SCCs (>3) has significantly decreased in H1 and mof double knockdown flies compared with H1 and GFP double knockdown flies. *n* = 66, 85 and 78 for the three groups, respectively. Germaria were dissected from 3 day old adult flies raised at 25°C (a-b), or at 29°C (c-f). Scale bars: 10 μm.
